# Using irregularly spaced current peaks to generate an isolated attosecond X-ray pulse in free-electron lasers

**DOI:** 10.1107/S1600577516013345

**Published:** 2016-10-06

**Authors:** Takashi Tanaka, Yong Woon Parc, Yuichiro Kida, Ryota Kinjo, Chi Hyun Shim, In Soo Ko, Byunghoon Kim, Dong Eon Kim, Eduard Prat

**Affiliations:** a RIKEN SPring-8 Center, Koto 1-1-1, Sayo, Hyogo 679-5148, Japan; b Physics Group, PAL-XFEL, Pohang Accelerator Laboratory, Pohang 37673, Republic of Korea; c Department of Physics, Center for Attosecond Science and Technology, Pohang University of Science and Technology, Pohang, Gyeongbuk 37673, Republic of Korea; d Max Planck Center for Attosecond Science, Max Planck POSTECH/KOREA Res. Init., Pohang, Gyeongbuk 37673, Republic of Korea; e Paul Scherrer Insitut, 5232 Villigen PSI, Switzerland

**Keywords:** free electron laser, attosecond pulse, terawatt power level

## Abstract

A method is proposed to generate an isolated attosecond X-ray pulse in free-electron lasers, using irregularly spaced current peaks induced in an electron beam through interaction with an intense short-pulse optical laser. In comparison with a similar scheme proposed in a previous paper, the irregular arrangement of current peaks significantly improves the contrast between the main and satellite pulses, enhances the attainable peak power and simplifies the accelerator layout. Three different methods are proposed for this purpose and achievable performances are computed under realistic conditions. Numerical simulations carried out with the best configuration show that an isolated 7.7 keV X-ray pulse with a peak power of 1.7 TW and pulse length of 70 as can be generated. In this particular example, the contrast is improved by two orders of magnitude and the peak power is enhanced by a factor of three, when compared with the previous scheme.

## Abstract 2

A method is proposed to generate an isolated attosecond X-ray free-electron laser pulse with the peak power beyond 1 TW.

## 1. Introduction   

Controlling the laser pulse length, or, more specifically, generating intense attosecond X-ray pulses, has been one of the most important technical challenges in X-ray free-electron lasers (XFELs) based on self-amplified spontaneous emission (SASE). Such XFEL pulses make it possible to observe ultrafast phenomena that are too fast to be investigated by conventional lasers (Bostedt *et al.*, 2013 ▸), although they have yet to be realised. In order to tackle this issue, many proposals have been made up to now, which can be divided into two types: with (Zholents & Fawley, 2004 ▸; Zholents, 2005 ▸; Saldin *et al.*, 2004*a* ▸,*b* ▸, 2006 ▸; Zholents & Zolotorev, 2008 ▸; Ding *et al.*, 2009 ▸; Xiang *et al.*, 2009 ▸; Zholents & Penn, 2010 ▸) and without (Emma *et al.*, 2004 ▸; Reiche *et al.*, 2008 ▸; Prat & Reiche, 2015 ▸; Prat *et al.*, 2015 ▸) an external optical laser. The accelerator layout to realise the former schemes may be usually more complicated than the latter; however, it offers an opportunity to synchronize accurately the pump laser and probe X-ray pulse for time-resolved experiments.

In a previous paper (Tanaka, 2013 ▸), a scheme was proposed to effectively compress the XFEL pulse by more than two orders of magnitude. It is an extension of the enhanced SASE (Zholents, 2005 ▸) (ESASE) scheme and is referred to as XFEL pulse compression (XFELPC), which is based on selective amplification of a specific X-ray pulse (main pulse) among many X-ray pulses generated by regularly spaced current peaks induced by the ESASE process.

Although the proposed XFELPC scheme offers an option to generate attosecond XFEL pulses with terawatt levels, there exists a critical problem to be solved for practical applications; many satellite pulses with non-negligible intensity appear ahead of the main pulse with a spacing of the wavelength of the ESASE laser, and thus the XFEL pulse generated by this scheme is regarded as an attosecond pulse train (APT), but not a single isolated attosecond pulse (IAP).

It is well known that the extreme-ultraviolet pulses, which are produced by a high-power optical laser focused onto a gas medium *via* the high-harmonic generation process, forms a train of attosecond pulses separated by one half cycle of the optical laser. Although there are several experiments taking advantage of APTs, most applications require intense IAPs. It should be noted, however, that generation of IAPs is much more challenging than that of APTs, because it requires a much more complicated laser system (Sansone *et al.*, 2006 ▸). This is the reason why a lot of effort has been devoted to generating intense IAPs (Goulielmakis *et al.*, 2008 ▸; Mashiko *et al.*, 2008 ▸; Ferrari *et al.*, 2010 ▸; Takahashi *et al.*, 2013 ▸) in the field of attosecond science.

The above discussion also applies to XFELs, and thus it is critically important to eliminate the satellite pulses in the XFELPC scheme and generate IAPs for practical applications. It should also be emphasized that the intense satellite pulses reduce the FEL gain of the main pulse, and thus the attainable peak power is lower than what is theoretically expected. In this paper, we propose a method to suppress the growth of satellite pulses and enhance the peak power of the main pulse, toward realisation of IAPs reaching terawatt levels. It also simplifies the accelerator layout in comparison with the original XFELPC scheme.

## 2. Issues on the original XFELPC scheme   

Let us first explain the problem of the original XFELPC scheme mentioned above in more detail. To illustrate it using a particular example, we carried out numerical simulations to compute the performance of XFELPC with the parameters summarized in Table 1 ▸. The beam current assumed here is relatively lower than what is actually achieved in existing XFEL facilities such as LCLS (Emma *et al.*, 2010 ▸) and SACLA (Ishikawa *et al.*, 2012 ▸). This is to relax the requirement on the tolerance of timing jitter between the electron bunch and ESASE laser pulse, which will be discussed later.  

**Figure 1:**
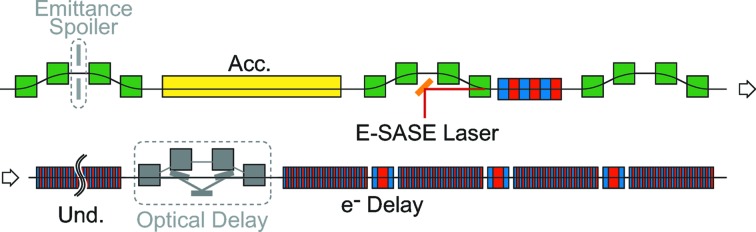
Accelerator layout for the original XFELPC scheme. Note that the two components shown in gray, the emittance spoiler and optical delay chicane, can be omitted in the new scheme to be discussed later.

Fig. 1 ▸ shows a schematic illustration of the accelerator layout to realise the original scheme. The emittance spoiler, or the slotted foil (Emma *et al.*, 2004 ▸), inserted into the bunch compressor section, defines the lasing domain in the electron beam. The optical delay chicane inserted between undulator segments adjusts the timing between the X-ray pulse and electron beam to initially pick up the main pulse. Note that these two components can be omitted in the new scheme, which will be described later.

**Figure 2:**
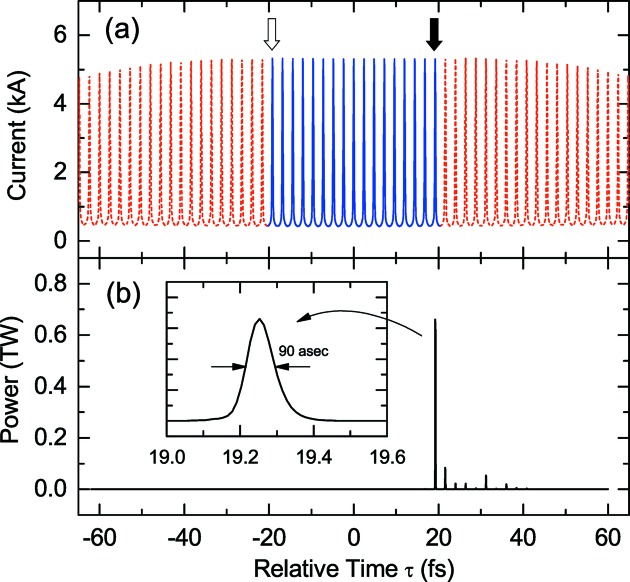
Estimated performances of the original XFELPC scheme: (*a*) current profile of the electron beam with the periodic current enhancement, and (*b*) XFEL pulse temporal profile at the end of the undulator beamline. In both figures, the head is to the right.

Fig. 2(*a*) ▸ shows the computed current distribution of the electron beam after the ESASE section. Note that the electron beam emittance in the region 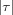 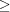 20 fs is assumed to be spoiled by the slotted foil. Here we introduced the relative time τ with respect to the center of the electron bunch. The red dashed line shows the current in the spoiled region where electrons do not contribute to lasing, while the blue solid line shows that in the lasing domain. The solid arrow indicates the head current peak that will generate the main pulse, while the empty arrow indicates the tail peak which will first amplify the main pulse after it is delayed by the optical chicane.

Using the current profile explained above, we carried out FEL simulations with *SIMPLEX* (Tanaka, 2015 ▸), under an assumption that the electron beam is injected to an undulator line composed of 24 segments, each of which has a magnetic length of 5 m, period of 18 mm and deflection parameter of 2.18, to generate XFEL pulses at the photon energy of 7.7 keV. The optical delay chicane is inserted after the sixth segment, while small magnetic chicanes to retard the electron beam are inserted at every drift section after the eighth segment, with the temporal delays being optimized to maximize the intensity of the main pulse.

Fig. 2(*b*) ▸ shows the typical temporal profile of an XFEL pulse at the end of the undulator beamline retrieved from the simulation results, with the inset showing the detail of the main pulse. Although the peak power reaches 0.7 TW with the FWHM pulse length shorter than 100 as, the main pulse is accompanied by a number of intense satellite pulses reaching 100 GW with a regular spacing of 2.4 fs corresponding to the wavelength of the ESASE laser. These satellite pulses can bring temporal ambiguity of the order of several femto­seconds, which is large enough to spoil the advantage of attosecond pulses in pump–probe experiments. In addition, they bring ambiguity to the estimated peak power of the main pulse, being given as the pulse energy divided by the typical pulse length. In this example, the partial pulse energy contained in the main pulse, which is given by integrating the radiation power over the corresponding temporal range of interest, is just 70% of the total pulse energy, meaning that the peak power may be overestimated by a factor of 1.5. What is more serious is that this percentage will fluctuate shot by shot. In many applications based on nonlinear X-ray optics, in which the signal intensity depends nonlinearly on the peak power of radiation, the accuracy and reliability of measurement may be significantly deteriorated by such an error.

## 3. Proposal to use irregularly spaced current peaks   

**Figure 3:**
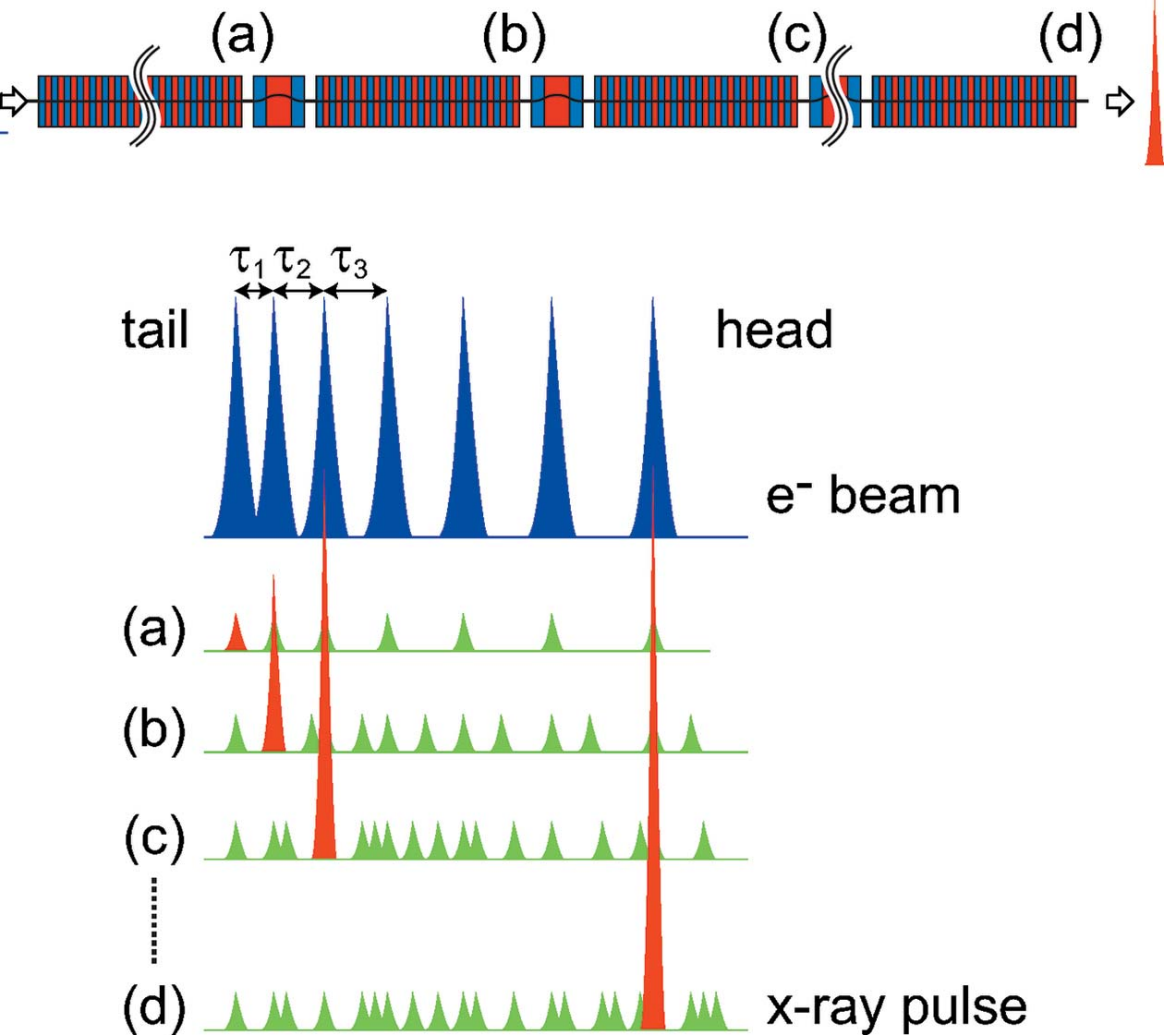
Selective amplification of the main pulse by the irregularly spaced current peaks.

The difficulties of the original XFELPC scheme explained above, *i.e.* intense satellite pulses intrinsic to the fundamental mechanism, can be completely solved if the current peaks induced in the electron beam through interaction with the ESASE laser are irregularly spaced. The principle is explained as follows with the assistance of Fig. 3 ▸, where the top figure shows a number of undulator segments with the electron delay chicanes in between, while the bottom schematically illustrates the temporal profiles of the electron beam injected into the undulator, and X-ray pulses at several locations indicated by (*a*)–(*d*). Note that the spacing between the *i*th and 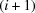
th current peaks denoted as 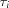 is not constant, and its variation rate should be optimized as discussed later.

At the position (*a*), the current peaks independently generate X-ray pulses in the normal SASE process, among which the X-ray at the tail end (painted red) works as the main pulse in the following process. After the electron beam is delayed by 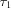 in the chicane, the main pulse coincides with the current peak just ahead of the tail peak, while the others do not, and thus only the main pulse is selectively amplified as shown in (*b*). Then the electron beam is delayed again by 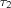, before the main pulse reaches saturation, or other X-ray pulses grow as intense satellite pulses, so that the selective amplification continues as shown in (*c*). This process can be repeated until the main pulse reaches the head current peak as shown in (*d*).

It is obvious that the two elements necessary in the original scheme, *i.e.* the slotted foil and optical delay chicane, can be eliminated in the new scheme. Instead of these elements, we need to modify the specifications of the ESASE laser and modulator to create current peaks with irregular spacings. This is not usually possible with the normal ESASE scheme based on the combination of a general optical laser and modulator and thus we propose three different methods as explained in the following sections.

It should be noted that all of them make use of an intense and ultrashort (∼fs) laser pulse as the ESASE laser, which necessarily enables an accurate temporal synchronization between the ESASE laser and X-ray pulse, as already mentioned in the original ESASE paper (Zholents, 2005 ▸).

### 3.1. Few-cycle pulse combined with a dedicated modulator   

In the first method, a few-cycle laser pulse interacts with an electron beam in a dedicated modulator, whose field amplitude is varied along the longitudinal axis. Let 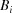 be the field amplitude of the modulator at the *i*th period. Then the slippage length 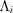 is given as
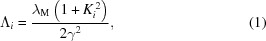
where 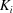 = 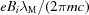 is the deflection parameter at the *i*th period, 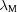 is the modulator period, *e* is the electron charge, *m* is the electron rest mass, and γ is the Lorentz factor of the electron beam. Because of the variation of the slippage length given above, the few-cycle laser pulse induces a chirped energy modulation in the electron beam, with the chirp rate dominated by 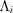, which is eventually converted to current peaks with the temporal spacing of 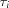 = 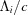. It should be emphasized that the ESASE laser pulse should be as short as possible, or at least shorter than a few cycles, otherwise the varying slippage results in reduction of the energy modulation, instead of generation of chirped modulation.

The variation of 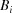 should be determined to satisfy two conditions. First, the variation rate should be large enough to avoid generation and amplification of satellite pulses adjacent to the main pulse. Second, the corresponding fundamental photon energy 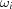 = 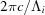 should be within the spectral bandwidth of the ESASE laser, where *c* is the speed of light; otherwise the interaction between the electron beam and ESASE laser becomes weaker, and thus the energy modulation is significantly reduced.

Let us consider the above two conditions with a particular example. We assume that an intense laser pulse with the central wavelength 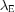 of 720 nm and FWHM pulse length of 3.7 fs (1.5 cycles) is injected into a modulator together with the electron beam assumed in the former example. It is worth mentioning that generation of such an ultrashort pulse with the pulse energy above 1 mJ has been reported (Park *et al.*, 2009 ▸).

The minimum variation rate of 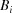 necessary to suppress the satellite pulses can be specified as follows. Let us consider the case when the main pulse is amplified by the 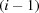
th current peak. Then an X-ray pulse may be generated by the *i*th current peak located 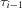 ahead of the main pulse, and can be an intense satellite in the following process. In order to disturb further amplification of this satellite pulse, the spacings between the current peaks should satisfy the condition 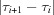 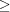 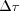, where 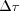 is the typical length of satellite pulses. Recalling that 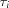 is roughly proportional to 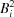 when the deflection parameter of the modulator is much higher than unity as in the present example, we have a criterion for 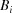,
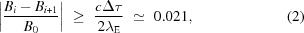
where we have substituted 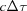 ≃ 30 nm, as found in Fig. 2(*b*) ▸. If this condition is satisfied, amplification of X-ray pulses that can potentially grow as intense satellite pulses can be avoided.

The second condition specifies the allowable maximum deviation of the field amplitude. Let us assume that the 1.5-cycle laser pulse is a Fourier-limited Gaussian pulse. The RMS bandwidth is then given as
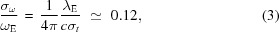
where 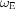 = 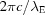 and 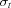 is the RMS pulse length. Recalling the relation between 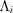 and 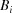, the above condition reduces to a criterion for the deviation of the field amplitude,
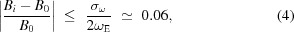
where 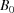 is the nominal field amplitude of the modulator.

Note that the maximum allowable field deviation 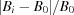 is inversely proportional to the number of cycles of the ESASE laser pulse, and fewer cycles allow for a larger deviation. In practice, it is reported that an intense monocycle pulse with a wavelength of 600 nm and pulse length of 2.1 fs (monocycle) has been generated (Wirth *et al.*, 2011 ▸). If we apply this monocycle pulse to the ESASE laser, the above criterion reduces to 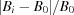 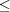 0.09. It should be noted, however, that generation of such a monocycle pulse requires a complicated system to synthesize the infrared, visible and ultraviolet light fields. Although the generated monocycle pulse has been successfully applied to probing the ultrafast dynamics, we are not sure if this scheme can be applied to the ESASE laser, which should be quite stable in terms of the temporal jitter and pointing stability. This is the reason why we have chosen the 1.5-cycle laser pulse for the proposed scheme, in which case the condition (4) should be satisfied.

**Figure 4:**
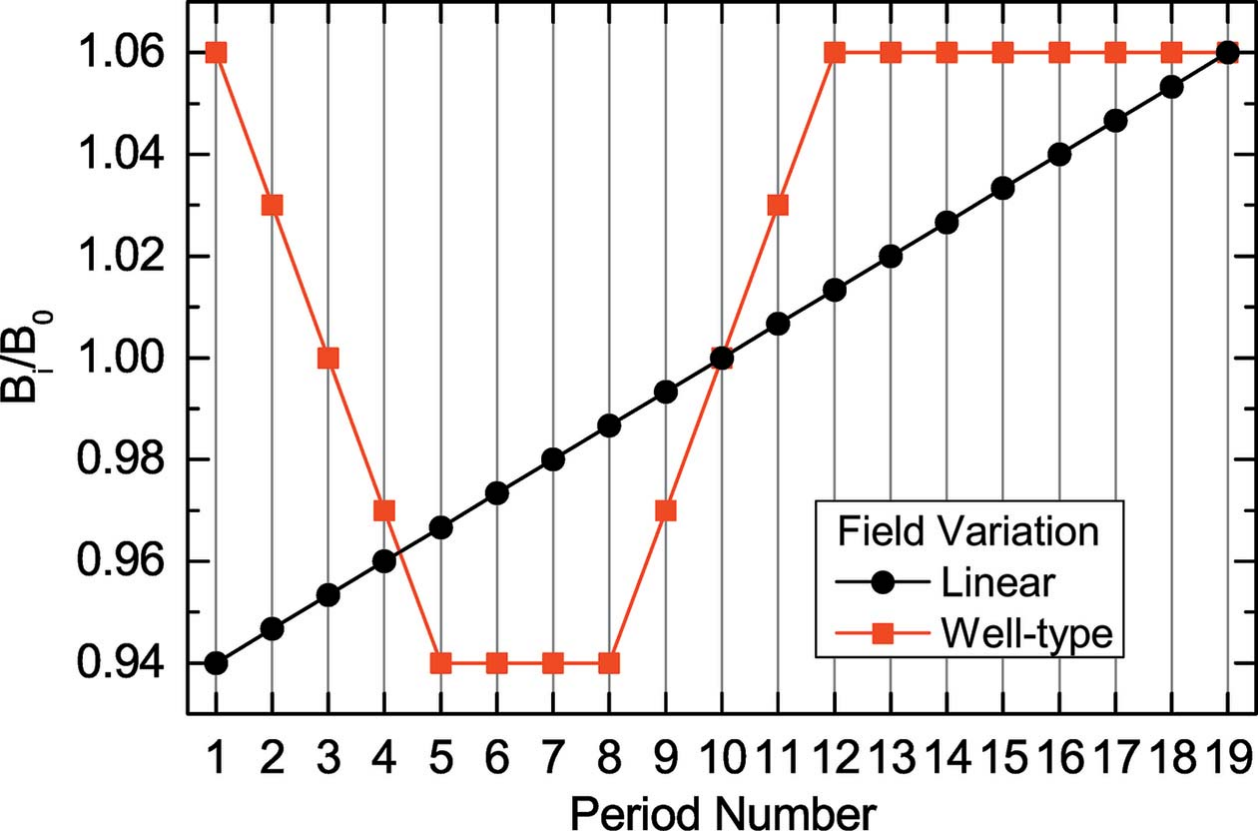
Variation of the field amplitude along the modulator axis for two taper types assumed to produce irregularly spaced current peaks.

Now let us optimize the variation of 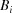 to suppress the satellite pulses. To create a sufficient number of current peaks to be consistent with the result for the original scheme as shown in Fig. 2(*b*) ▸, we need 19 modulator periods. If we assume a simple linear taper as the variation of 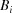, which is the most straightforward one, we have a profile of 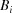 as shown by the black circles in Fig. 4 ▸. It is obvious that the linear taper does not satisfy the condition (2), as long as the condition (4) is kept.

Instead of the linear taper, which may not work in the present example as mentioned above, we propose to use an alternative field profile shown by the red squares in Fig. 4 ▸, which is referred to as a well-type profile and is composed of four regions: descending, flat-bottom, ascending and flat-top.

It is obvious that this profile satisfies the condition 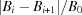 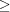 0.021 in the descending and ascending regions. The satellite pulses, which can possibly arise in the current peaks corresponding to the flat-bottom region, cannot grow as intense pulses, because the following ascending region suppresses the overlap between satellite pulses and current peaks. In addition, the intensity of satellite pulses generated by the current peaks corresponding to the flat-top region is expected to be much lower than that of the main pulse.

The slopes of the descending and ascending regions can be arbitrarily chosen as long as the condition (2) is satisfied; they can be asymmetric as well. As an example in this paper, we have chosen a symmetric profile with the variation rate of 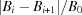 = 0.03. Besides the slope, we have another parameter to be optimized: the number of periods in the flat-bottom region, 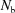, which has been assumed to be 3 in Fig. 4 ▸. In order to optimize 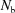, we need to perform FEL simulations to quantify the intensity of satellite pulses, which is to be discussed later.

To compute the energy modulation of the electron beam induced by the 1.5-cycle pulse in the modulator, and eventually the current profile after passing through an optimized chicane, we need to specify the waist size at the modulator and the pulse energy of the ESASE laser. The former is optimized so that the Rayleigh length is comparable with the modulator length, which roughly maximizes the interaction between the electron beam and ESASE laser; a larger waist size results in lower power density, while a smaller one results in larger diffraction effects, both of which will reduce the interaction efficiency.

With the above condition regarding the waist size, it has been found that current peaks around 10 kA can be generated if we assume that the pulse energy of 1.2 mJ is fully available. It should be noted, however, that such a high peak current requires a large energy modulation around 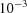 and the resultant space charge effects become serious, both of which may degrade the FEL gain. We assume to use the minimum pulse energy necessary to generate current peaks around 5 kA to avoid the above problems. Note that these criteria for the waist size and pulse energy are also applied to other different conditions to be discussed in later sections.

**Figure 5:**
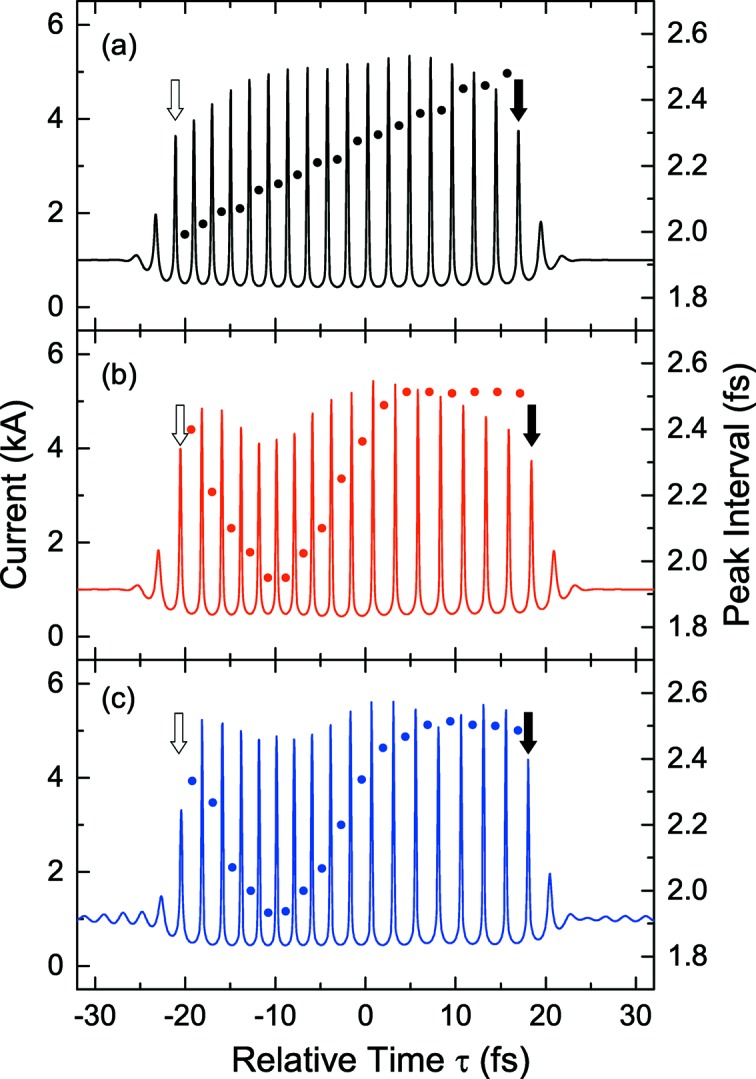
Computed current profiles of the electron beam with irregularly spaced current peaks generated in three different configurations: (*a*) 1.5-cycle laser pulse with the linearly tapered modulator; (*b*) same as (*a*) but with the well-type profile; (*c*) double-chirped pulse with the two-period modulator. The spacings between current peaks are also shown by solid circles in each figure.

Figs. 5(*a*) and 5(*b*) ▸ show the current distributions computed for two different modulators with the linear taper and well-type profile as specified in Fig. 4 ▸, respectively. The pulse energy is assumed to be 0.3 mJ in both cases. The spacings of current peaks are also indicated by solid circles in the same figures, showing a clear difference between the two modulator types. The head and tail current peaks are indicated by solid and empty arrows, respectively.

### 3.2. Double-chirped pulse combined with a few-period modulator   

The second method is based on a chirped pulse with an optimized chirp rate. It is easy to understand that the laser pulse, whose instantaneous wavelength varies like the well-type profile as shown in Fig. 4 ▸, can reproduce the current peaks shown in Fig. 5(*b*) ▸, if a few-period modulator is used.

**Figure 6:**
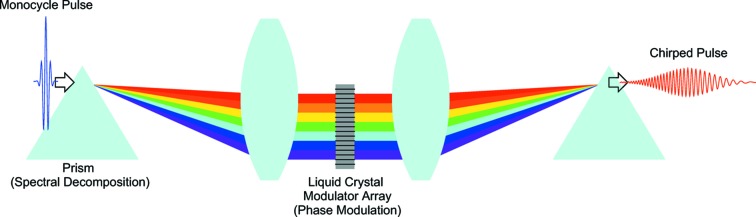
Schematic illustration of the laser optics to realise an arbitrary chirp rate.

In order to control the instantaneous wavelength as explained above, we propose to use a special optics as schematically illustrated in Fig. 6 ▸. After the laser pulse passes through a dispersive optical element for spectral decomposition, the spectral phase and intensity are modulated by means of a spatial light modulator (Weiner, 2000 ▸; Wilson *et al.*, 2007 ▸), which is then combined again to form a chirped pulse with an arbitrary chirp rate. It is worth mentioning that femtosecond pulse shaping with this method has been successfully demonstrated (Tanigawa *et al.*, 2009 ▸; Wang *et al.*, 2008 ▸) with a pulse energy at the millijoule level.

**Figure 7:**
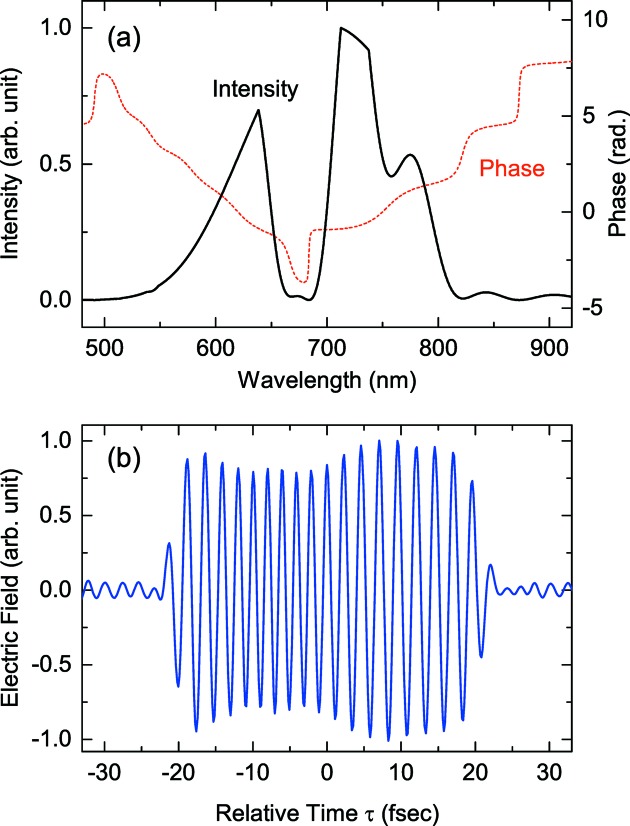
(*a*) Spectral phase and intensity modulation as a function of the wavelength. (*b*) Computed waveform of radiation field after the phase and intensity modulation.

As an example of the method explained above, we optimized the spectral phase modulation for the purpose of reproducing the current distribution shown in Fig. 5(*b*) ▸. The result is shown in Fig. 7(*a*) ▸, in which the phase and intensity modulation are plotted as a function of the wavelength. Fig. 7(*b*) ▸ shows the waveform of radiation field in the time domain computed with the parameters of the 1.5-cycle pulse assumed in the former example and the phase and intensity modulation shown in Fig. 7(*a*) ▸, which is hereinafter referred to as the double-chirped pulse.

We note that the pulse energy will be reduced in the above process for two reasons: throughput of the spatial light modulator and intensity loss due to the pulse shaping. The former is supposed to be 50%, while the latter is also estimated to be 50%, which can be actually computed from the required spectral profile as in Fig. 7(*a*) ▸, and the input spectral profile of the 1.5-cycle pulse. As a result, the input pulse energy should be four times larger than what is required for the ESASE process.

Fig. 5(*c*) ▸ shows the current distribution computed with the assumption that the electron beam and the double-chirped pulse generated in the above scheme are injected into a two-period modulator. In this example, the pulse energy and waist size are assumed to be 0.28 mJ and 0.37 mm, respectively. Considering the energy loss, we need an initial pulse energy of 1.1 mJ before the pulse-shaping process. The two current distributions shown in Figs. 5(*b*) and 5(*c*) ▸ agree reasonably well, except a small oscillation found in the tail and head part of the latter, which may have no impact on the performances of XFELPC.

### 3.3. Pulse stacking combined with a few-period modulator   

**Figure 8:**
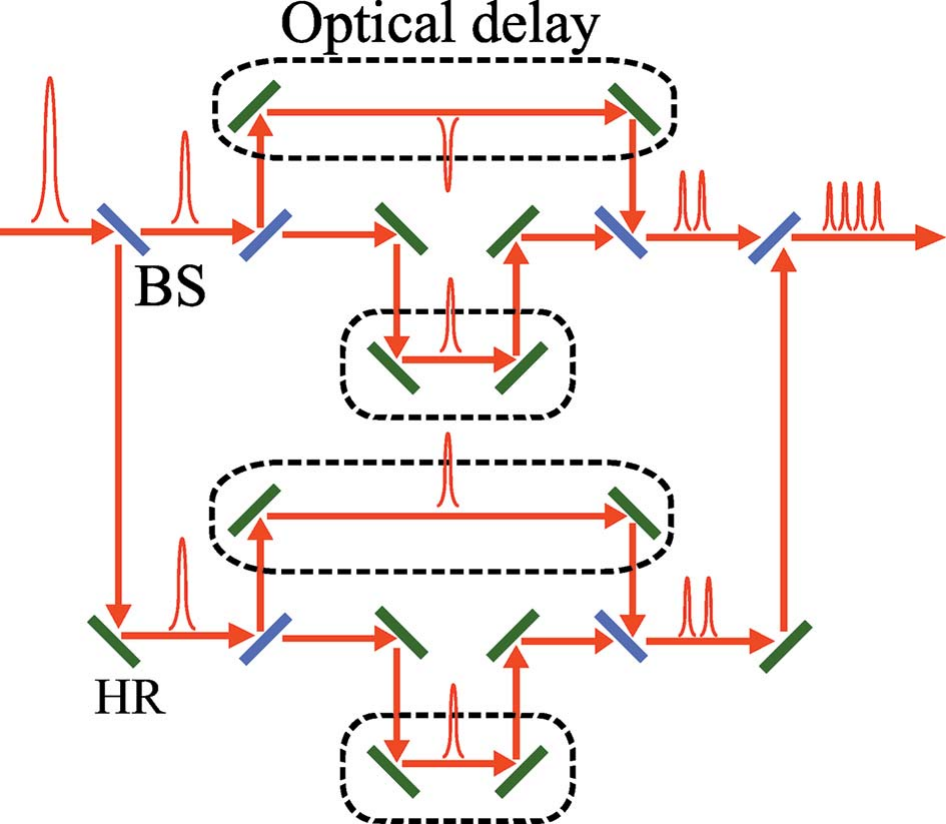
Production of a train of a few cycle laser pulses based on the pulse stacking method.

The third method is based on the pulse stacking concept, which has already been used in XFEL facilities to obtain a flat-top current profile of the electron beam (Hyun *et al.*, 2009 ▸). In this application, several laser pulses with the same spacing are stacked together to generate a flat-top laser pulse. This idea can be extended to make a train of a few-cycle laser pulses with different spacing values, by providing a different optical delay to each laser pulse. A schematic of the proposed design is shown in Fig. 8 ▸. We can control the arrival time of each laser pulse by using optical delays, illustrated by the dashed boxes in Fig. 8 ▸. These optical delays will eventually be used to control the spacing between the current peaks.

**Figure 9:**
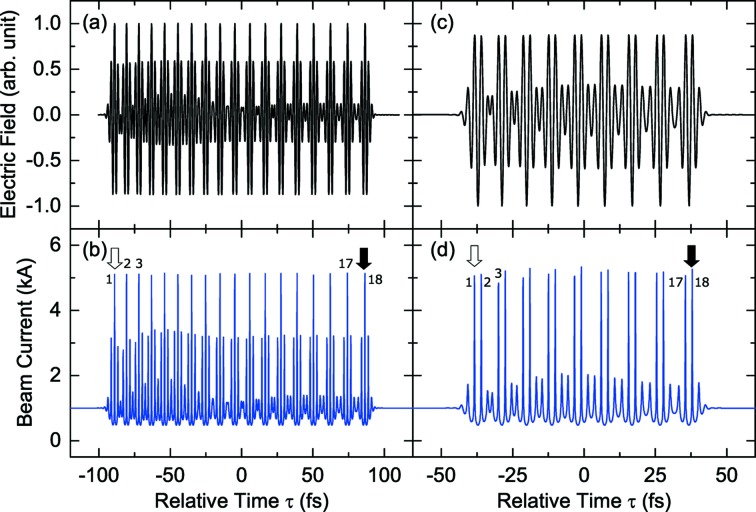
Temporal profiles of the radiation field generated by the pulse stacking method with (*a*) 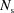 = 18 and (*c*) 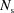 = 9, and corresponding current profiles (*b*) and (*d*), respectively.

The advantage of this method over the other two is that the temporal spacing can be in principle chosen arbitrarily. Fig. 9(*a*) ▸ shows an example of the radiation field generated by stacking the 1.5-cycle pulses with the wavelength of 720 nm. The number of stacked pulses (= 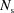) is 18 in this example. The optical delays have been adjusted so that the spacing 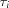 is given as 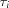 = 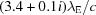. This is not only to satisfy the condition 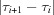 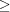 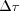 but also to avoid interference between pulses. The current profile to be generated using this radiation pulse together with the two-period modulator is shown in Fig. 9(*b*) ▸, where the head and tail current peaks are indicated by solid and empty arrows, respectively, together with numbers indicating the index *i*. The pulse energy and waist size are assumed to be 0.55 mJ and 0.37 mm, respectively. We find that 18 current peaks reaching 5 kA are arranged with the temporal spacings specified by 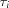, which can continuously and selectively amplify the main X-ray pulse by adjusting the electron delays accordingly. Note, however, that these current peaks are followed by pre- and post-peaks reaching 3 kA, and can generate intense satellite pulses.

Another example of pulse stacking is shown in Fig. 9(*c*) ▸, where nine pulses are stacked with the same spacing as those in the above. It should be noted that the carrier envelope phase is reversed to generate a pair of current peaks for each stacked pulse. Fig. 9(*d*) ▸ shows the current profile computed with the assumed pulse energy of 0.45 mJ. The current peaks are arranged with the spacing 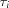 given as
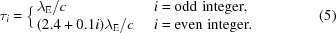
The satellite current peaks are much less significant in this case; however, half of the spacings have the same value of 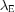, the effects of which are quantitatively discussed in the next section.

## 4. Estimation of the performance   

**Figure 10:**
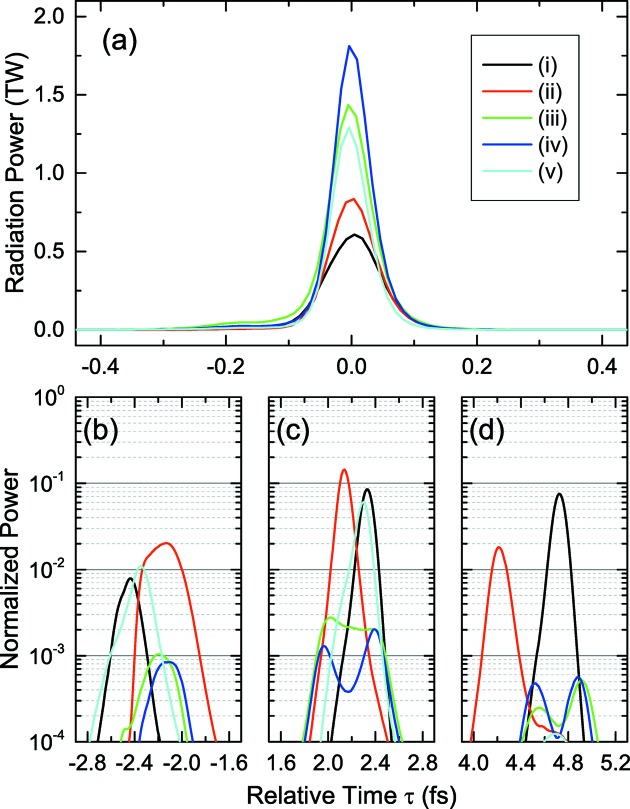
Averaged temporal profiles of (*a*) the main pulse and (*b*)–(*d*) satellite pulses normalized by the peak power, simulated with five different conditions (i)–(v). Refer to Table 2 ▸ for details of the simulation conditions.

Now let us show the results of simulations carried out to examine the performances of XFELPC with the irregularly spaced current peaks, in comparison with the original scheme. The optical chicane after the sixth undulator segment, assumed in the original scheme to initially pick up the main pulse, is replaced with a small magnetic chicane to retard the electron beam in the new scheme. In order to eliminate the shot-to-shot fluctuations intrinsic to the SASE process, we repeated simulations 30 times for a specific condition with different random seeds, retrieved the temporal profiles of XFEL pulses, and computed their average, shown in Figs. 10(*a*)–10(*d*) ▸ for five representative simulation conditions. Note that Fig. 10(*a*) ▸ shows the main pulse, while the others show satellite pulses normalized by the peak power.

Table 2 ▸ summarizes the conditions assumed in the simulations, and their results in terms of the peak power of the main and first satellite pulses, and the FWHM pulse length. Note that the average (*A*) and standard deviation (σ) are evaluated from the results of 30 simulations, which are given in the format of 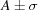. The ‘Profile plot’ columns indicate the figure numbers showing the temporal profiles of the electron beam and XFEL pulse. For example, those for the original scheme are shown in Fig. 2(*a*) ▸ and Figs. 10(*a*)–10(*d*)-(i), respectively.

Among all the conditions examined above, the double-chirped pulse configuration, whose XFEL profiles are shown by the blue lines in Figs. 10(*a*)–10(*d*), gives the best results in terms of both the peak power and contrast. In comparison with the original scheme, the peak power is enhanced by a factor of three, and the contrast is enhanced by nearly two orders of magnitude. We also note that the partial pulse energy contained in the main pulse is as high as 98% of the total pulse energy, and thus the peak power estimation will be much more reliable.

It should be noted that the above result is not universal; the best configuration depends on the conditions such as the number of undulator sections, electron beam parameters and ESASE laser specifications. The double-chirped pulse configuration, which gives the best result in the particular example described above, may not be necessarily the best for other conditions.

## 5. Tolerance of timing jitter   

Apart from the advantages of using the irregularly spaced current peaks described in the previous section, there exists a critical problem to be solved so that this scheme can be actually put into practical use: the timing jitter between the ESASE laser pulse and electron beam. It is obvious that the current peaks should be located well within the electron bunch so that the XFELPC scheme works effectively. It is thus important to quantitatively investigate the effects due to the relative timing shift 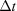 of the ESASE laser pulse with respect to the electron beam.

**Figure 11:**
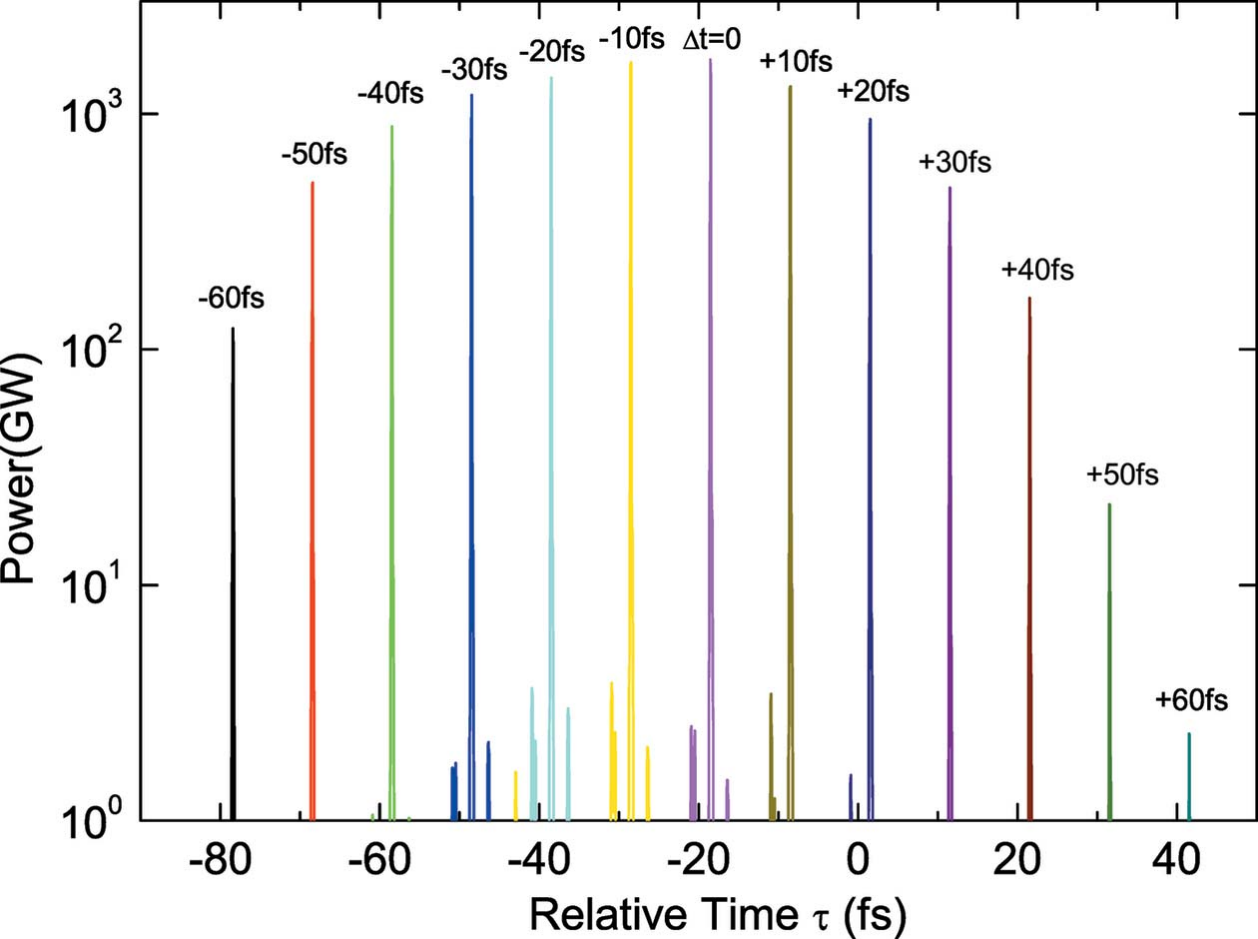
Averaged temporal profiles of XFEL pulses simulated with the double-chirped pulse method for different conditions of temporal synchronization.

As a simple example, we computed the temporal profiles of attosecond pulses generated by the XFELPC scheme for different conditions of temporal synchronization, under an assumption that an electron bunch having a Gaussian profile with the FWHM bunch length of 150 fs interacts with the double-chirped pulse. The results are plotted in Fig. 11 ▸, in which 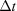 = 0 means that the center of the chirped pulse coincides with that of the Gaussian electron bunch. We note that a relatively long bunch length of 150 fs has been assumed here, which is validated by the low beam current of 1 kA.

We find that the attosecond pulse generated by the XFELPC scheme shifts according to 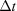, with the peak power reduction roughly symmetric with respect to the condition 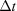 = −10 fs. It should be noted that, even with a large timing shift of 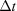 = −10 ± 30 fs, the peak power roughly reaches 1 TW. What should be emphasized more is that isolated attosecond pulses with peak power around 100 GW can still be generated with a pulse length less than 100 as, even in the worse condition of 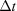 = −10 ± 50 fs.

Summarizing the above simulation results, it is reasonable to say that the tolerance of timing jitter in the conditions under consideration is, roughly speaking, ±40 fs. It is out of the scope of this paper to discuss the feasibility of the timing system to realise the synchronization within this tolerance. Instead, we mention the useful information found in the paper by the DESY group, in which the timing jitter between the XFEL and optical laser pulses has been measured to be 33 fs RMS (Schulz *et al.*, 2013 ▸). Although it is slightly lower than the tolerance mentioned above, this timing jitter may not be small enough for the proposed XFELPC scheme to be fully functional. To be specific, the peak power can fluctuate from shot to shot.

## 6. Summary   

We have proposed to use irregularly spaced current peaks to suppress the intense satellite pulses intrinsic to the original XFELPC scheme, and enhance the peak power of the main pulse, for the purpose of generating IAPs in the real sense of the term. Three methods have been described to generate a desired current profile, and the double-chirped pulse configuration has been found to be the best under the conditions considered in this paper.

In the simulations carried out to estimate the expected performances, we assumed relatively low beam current to relax the tolerance of timing jitter. It is worth mentioning that, if the timing jitter can be reduced further, we can compress the electron bunch more to enhance the electron beam current before interaction with the ESASE laser, in which case the achievable peak power will be further enhanced.

## Figures and Tables

**Table 1:** Parameters of the electron beam, modulator and ESASE laser assumed to compute the performance of the original XFELPC scheme

Electron beam	Energy	7 GeV
	Normalized emittance	0.4 µm
	Energy spread	8 × 10^−5^
	Beam current	1.0 kA

Modulator	Period length	30 cm
	Period number	15
	Peak field	1.5 T

ESASE laser	Wavelength	720 nm
	Pulse energy	0.4 mJ
	Pulse length (FWHM)	250 fs

**Table 2:** Summary of simulations carried out to examine the performances of the new scheme in comparison with the original scheme

			Profile plot		Peak power		
Simulation condition			e^−^	XFEL	Main (TW)	Satellite (%)	Pulse length (as)
Original scheme			2(*a*)	(i)	0.61 ± 0.41	8.5 ± 6.5	84 ± 14
1.5-Cycle pulse	Linear taper		5(*a*)	(ii)	0.83 ± 0.47	14 ± 14	74 ± 9.0
	Well-type	*N*_b_ = 1	NA	NA	1.5 ± 0.56	0.66 ± 0.47	66 ± 3.0
		*N*_b_ = 3	5(*b*)	(iii)	1.4 ± 0.49	0.27 ± 0.35	69 ± 2.3
		*N*_b_ = 5	NA	NA	1.4 ± 0.35	1.1 ± 0.89	68 ± 2.5
Double-chirped pulse			5(*c*)	(iv)	1.8 ± 0.45	0.20 ± 0.16	62 ± 3.0
Pulse stacking	*N*_s_ = 18		9(*c*)	NA	1.6 ± 0.36	30 ± 9.1	62 ± 1.5
	*N*_s_ = 9		9(*d*)	(v)	1.3 ± 0.45	6.1 ± 4.8	59 ± 3.5
